# The Potential Role of Probiotics in the Management of Childhood Autism Spectrum Disorders

**DOI:** 10.1155/2011/161358

**Published:** 2011-10-26

**Authors:** J. William Critchfield, Saskia van Hemert, Michael Ash, Linda Mulder, Paul Ashwood

**Affiliations:** ^1^Department of Medical Microbiology and Immunology, University of California, Davis, CA 95616, USA; ^2^R&D Department, Winclove Bio Industries, Hulstweg 11, 1032 LB Amsterdam, The Netherlands; ^3^Integrative Health Consulting, Milber, Newton Abbot, Devon TQ12 4SG, UK

## Abstract

Gastrointestinal (GI) dysfunction has been reported in a substantial number of children with autism spectrum disorders (ASD). Activation of the mucosal immune response and the presence of abnormal gut microbiota are repeatedly observed in these children. In children with ASD, the presence of GI dysfunction is often associated with increased irritability, tantrums, aggressive behaviour, and sleep disturbances. Moreover, modulating gut bacteria with short-term antibiotic treatment can lead to temporary improvement in behavioral symptoms in some individuals with ASD. Probiotics can influence microbiota composition and intestinal barrier function and alter mucosal immune responses. The administration of probiotic bacteria to address changes in the microbiota might, therefore, be a useful novel therapeutic tool with which to restore normal gut microbiota, reduce inflammation, restore epithelial barrier function, and potentially ameliorate behavioural symptoms associated with some children with ASD. In this review of the literature, support emerges for the clinical testing of probiotics in ASD, especially in the context of addressing GI symptoms.

## 1. Introduction

Autism spectrum disorders (ASD) are serious developmental disorders with soaring prevalence, affecting 1 in 91 children in the United States [1]. ASD are characterised by a spectrum of symptoms, including decreased verbal skills and social withdrawal, repetitive behavior, insistence to routines, and unusual response to sensory stimuli. Available treatments currently include specialized behavioral interventions [2] and pharmacologic interventions to assist in comorbid or associated symptoms [3]. With the underlying causes of ASD still largely undefined, the etiology of ASD is currently the focus of intensive study, with particular areas of interest including the role of environmental, genetic, and epigenetic factors [4, 5]. These efforts share a common goal of developing new insights which can be translated into novel, effective strategies for the prevention and treatment of ASD.

The diagnosis of ASD is reached by observation of behavior and the application of well-characterized instruments, such as the Autism Diagnostic Interview-Revised (ADI-R), and the Autism Diagnostic Observation Schedule-Generic (ADOS-G). There is great heterogeneity in the individuals that receive the ASD diagnosis. This heterogeneity is not necessarily due to the diagnostic instruments but because the behaviors displayed by a group of individuals with ASD likely arise from a variety of causes. However, the causes of ASD are largely unknown, except in the rare instances in which identifiable genetic abnormalities give rise to a syndrome with ASD features. Experimental approaches seeking to obtain new insights into the etiology of ASD are increasingly focused on the selection of subgroups of ASD individuals who share a defining group of characteristics. By taking this approach, it is possible that specific factors associated with certain aspects of ASD can more readily be identified. For example, a prominent subgroup of individuals with ASD suffers from gastrointestinal (GI) dysfunction that stems from yet unknown causes. 

 The prevalence of GI symptoms in ASD is substantial, with common syptoms including diarrhea, constipation, vomiting/reflux, abdominal pain/discomfort, gaseousness, and unusually foul-smelling stools [6–8]. Individuals with ASD who have GI symptoms, compared to those without GI symptoms, often display significantly higher measures of irritability, anxiety, and social withdrawal [8]. While these studies illustrate that GI symptoms are present, the pathophysiology of these symptoms is not well understood. Despite the current lack of evidence surrounding a known GI pathology in ASD, almost one-fifth of the physicians encourage the use of probiotics for children with ASD, according to a recent survey in the United States [9]. In addition, almost 60 percent of the physicians accept the use of probiotics when the family is already utilizing them. Thus, the purpose of this paper is to put forth whether there is a case for clinical testing of probiotic preparations in selected groups of children with ASD. Rationale will be presented for the relevance of probiotic use not only in groups with GI symptoms but also in light of other immunologic and intestinal abnormalities identified in the ASD population.

Probiotics are living nonpathogenic microorganisms, which beneficially affect the host's health, when administered in adequate amounts as food ingredient or supplement. Probiotic products consist mainly of lactic acid-producing bacteria, such as lactobacilli, lactococci, and bifidobacteria, or yeasts such as *Saccharomyces boulardii*. The well-established health effects of probiotics vary with the species and particular strain(s) of bacteria chosen, based on genetic differences and the nature of bacteria-host interactions [10]. Probiotics have been used safely in the fermentations of food products for decades. Therefore, the United States Food and Drug Administration has designated probiotics as Generally Recognized as Safe (GRAS), and the European authorities provide a list of bacterial species with a Qualified Presumption of Safety (QPS). 

Research on probiotics has shown efficacy of probiotic bacteria in a wide variety of health problems. Gastrointestinal difficulties such as antibiotic-associated and acute infectious diarrhea, inflammatory bowel disease (IBD), and irritable bowel syndrome (IBS) have been shown to be responsive to probiotics. In addition, probiotics have also shown activity in influencing the host's immune system [11, 12]. With the growing research on probiotics and the integral part they play in the health of not just the GI tract but the body as a whole, their inclusion as an integrative treatment may be of use for children with ASD. 

## 2. The GI Tract in ASD

A notable proportion of children with ASD suffer from gastrointestinal (GI) symptoms stemming from unknown causes. The precise prevalence of GI symptoms in children with autism is not clear, with estimates ranging from between 9 to 70% [13]. The discrepancy of figures may reflect the studies reporting these estimates and range from lower estimates in some population studies to higher estimates seen in specialized GI clinics. Moreover, the assessment of GI symptoms and what constitutes GI symptoms accounts for much variation in these estimates. The fact that GI symptoms are a significant problem in autism is not in dispute but is often understated. Since GI symptoms in ASD may be important contributors to behavioral difficulties [13], carefully designed studies that investigate the full range of symptoms described for this ASD cohort are required to establish prevalence values with greater confidence. In agreement with the idea that the severities of GI symptoms and autistic symptoms could possibly be linked, Nikolov et al. reported that children with ASD and GI symptoms, compared to those without GI symptoms, display significantly higher measures of irritability, anxiety, and social withdrawal [8]. More recently, Adams et al. studied 58 children with ASD, measuring both GI symptoms and the severity of autism [14]. Their data showed a strong positive association of autism severity with GI dysfunction. This association was evident within each of the subcategories of their autism assessment, including speech, social, sensory/cognitive, and physical/behavioural. This important new finding, while not defining a cause-effect relationship, nevertheless emphasizes the importance of developing new approaches to relieve GI symptoms in children with autism.

The GI symptoms associated with ASD are remarkably similar to those of irritable bowel syndrome (IBS) which affects 10–20% of the US population. IBS is characterized by abdominal pain/discomfort which sometimes improves upon defecation and is associated with changing stool frequency and/or changing stool consistency [15]. In a given individual, constipation or diarrhea (or neither) may predominate. Other symptoms which can accompany IBS (as well as ASD) include bloating, excess gas, gastric reflux, and food sensitivities. A careful evaluation of the patient as set forth in the ROME III criteria for functional bowel disorders [15] provides the diagnosis of IBS; this approach derives from the fact that, like ASD, there is no known biological signature or biomarker for IBS. In another parallel to ASD, IBS patients commonly have extraintestinal comorbid conditions, some of which suggest a CNS component. These include sleep difficulties, anxiety, depression, headache, and fatigue [16, 17]. Due to the similarity of GI symptoms between ASD and IBS, an established IBS symptom scoring system has been employed in the assessment of GI symptoms in ASD [18].

Based on possible similarities between IBS and GI symptomatology in ASD, it is pertinent to note the recent successes in the treatment of IBS with probiotics. Specifically, a recent meta-analysis [19] of clinical trials highlighted two well-designed studies reporting that *Bifidobacterium infantis* can significantly reduce GI symptoms in IBS. In the first study, 77 subjects with IBS were evaluated over a 4 week period for GI symptoms and stool patterns, then randomized to one of three blinded treatments: *Lactobacillus salivarius*, *Bifidobacterium infantis* 35624, or placebo [20]. Symptoms including distention/bloating, difficulty with bowel movements, and abdominal discomfort/pain dropped significantly in the *B. infantis* group, compared to placebo, during the subsequent 8 week intervention period. These changes took place quickly, being evident by the end of the first week of treatment and essentially maximal by two weeks. In a larger multicenter follow-up study focusing solely on *B. infantis *35624, 362 subjects recruited in a primary care setting underwent a two-week baseline observation period then randomization to one of three encapsulated doses of *B. infantis* or placebo for a six-week intervention period [21]. Confirming and extending results of the first study, treatment with *B. infantis* (10^8^ cfu/day) was associated with marked improvements such as lessening of (1) bloating/distention, (2) abdominal pain/discomfort, (3) passage of gas, (4) straining with defecation, and (5) sense of incomplete evacuation. Other improvements included scores for (1) bowel habit satisfaction and (2) global self-assessment of IBS relief. The improvements in this study were observed after approximately four weeks, in contrast to two weeks in the first study, with the slower response attributed to the encapsulated formulation which may be a less-efficient delivery system. In conclusion, clear parallels between GI symptoms in ASD and IBS, along with successes in treating IBS symptoms with probiotics, make it clear that clinical trials with probiotics in children with ASD who have GI symptoms are warranted.

## 3. Microbiota Abnormalities

Some studies suggest that children with ASD have disturbances of their intestinal microbiota compared to typically developing children. In the feces of children with ASD versus healthy controls, 10-fold higher levels of *Clostridium* species were reported, with a greater diversity observed in the *Clostridium* species [22]. However, a comparison group consisting of ASD patients without GI symptoms was not included, so the relationship between *Clostridium* species and GI symptoms could not be clarified. A subsequent study revealed that there were elevations in three different *Clostridium* groups; *Clostridium bolteae* and *Clostridium* clusters I and XI, in individuals with ASD, but unfortunately this study did not report on the frequency of GI symptoms in the subjects [23]. More recently, Parracho et al. [24] showed that the fecal content of the *Clostridium histolyticum* group, known toxin producers [25], was elevated in ASD children compared to healthy unrelated controls but not compared to healthy siblings. The study included children with ASD with and without GI symptoms and found that within the ASD group high levels of *Clostridium* species were significantly associated with GI problems. Moreover, in a recent pyrosequencing-based study, multiple differences in fecal microbiota composition were observed among children with ASD and typically developing controls [26]. These changes included shifts at the phylum level toward a higher proportion of *Bacteroidetes* and a lower level of *Firmicutes* in autism. At the genus level, several *Bifidobacterium* species were lower in autism while *Desulfovibrio* genus was more highly represented. In general, the sibling control group was similar to the ASD group, and it was suggested that this could result from the transfer of microbial species from ASD children to unaffected siblings and may represent a risk factor for ASD rather than the result of ASD pathology. A recent culture-based assessment of fecal species in autistic children supports the finding of lower *Bifidobacteria *levels [14]. Finally, metabolic phenotypes, some of which can be influenced by shifts in gut microbial populations, can now be assessed via measurement of key metabolites in urine. In the context of autism, Yap et al. have defined intriguing urinary changes related to amino acid and microbial metabolites [27]. The relationship, if any, of these altered patterns to the gut microbiota in autism remains to be established.

The hypothesis that a gut microbial imbalance, such as the presence of toxin-producing *Clostridium species*, could contribute to ASD behavioral symptoms was addressed in a small study consisting of 11 children with chronic diarrhea and a late-onset phenotype of ASD [28]. Subjects were treated for 8 weeks with vancomycin, a strategic drug in the treatment of chronic *Clostridium difficile *diarrhea. Scores for behavior and communication improved significantly during the treatment period; however, these gains in behavior were temporarliy and deteriorated in most cases after termination of the treatment. Unfortunately, GI symptomatology and fecal flora composition were not reported, and the experimental design suffered from a lack of a control group. In spite of these shortcomings, this study is the only one of its kind and suggests that ASD symptoms in a selected group of children may have a relationship to the composition of intestinal bacterial species that are sensitive to vancomycin. 

Disruption of the balance that exists between different enteric microbiota populations may predispose an individual to altered gut motility and secretion, which results in diarrhea or constipation, symptoms commonly reported in autistic patients. These changes are, in turn, likely to influence the balance of enteric microbiota. Probiotics can be useful for maintaining or restoring the balance of the intestinal microbiota [29]. For example, a probiotic mixture has been shown to affect both the composition as well as the metabolic activity of the fecal microbiota in healthy volunteers taking the antibiotic amoxicillin [30]. In addition, specific strains of probiotics have been shown to inhibit the growth of different *Clostridium* species [31–34]. There is also good *in vivo* evidence that probiotics can be useful for the treatment of *Clostridium difficile *infections (for a review, see [35]). By analogy, the testing of probiotics in ASD could reveal whether they are able to normalize gut microbial populations, reduce GI symptoms (in those affected), and alleviate behavioral difficulties.

## 4. Mucosal Immunity

Several studies have sought to determine whether there is evidence of intestinal pathology in ASD. Findings of significant but subtle widespread gastrointestinal pathology have been noted in children with ASD who have GI symptoms sufficient to warrant clinical followup [6, 13, 36]. However, the existence of a gastrointestinal pathology specific to persons with ASD is a controversial topic. Histology, immunohistochemistry, and flow cytometry evidence has consistently shown a subtle, panenteric infiltration of immune cells such as lymphocytes, monocytes, NK cells, and eosinophils into the walls of the GI tract in some children with ASD, compared with typically developing children with GI symptoms [37–42]. Furthermore, the infiltrating lymphocytes exhibit a marked proinflammatory phenotype with increased CD3^+^ TNF*α* ^+^ cells, CD3^+^ IFN*γ* ^+^ cells and reduced regulatory CD3^+^IL-10^+^ cells in ASD children with GI symptoms [38, 39]. The observatons, thus far made, do not suggest that any pathology is similar to known inflammatory conditions such as Crohn's disease, ulcerative colitis, or celiac disease. Furthermore, intestinal immunological abnormalities can also be found in children with typical development, as well as children with food allergies and immunodeficiencies [13]. By using two inflammatory markers in the feces, which are increased in some IBD patients, there was no evidence found for a link between active (i.e., neutrophil related) inflammation in the gut and autism [43]. Thus, despite evident symptomatology there remains significant contention as to whether this relates to GI pathophysiology in ASD, with many proponents and detractors [44]. 

Healthy individuals have an intact intestinal barrier, essential in maintaining health and preventing tissue injury [45]. Despite the arguments surrounding gut pathology in ASD, several studies have shown that the integrity of the intestinal mucosal barrier might be compromised in ASD [36]. The intestinal barrier quality can be measured by urinary levels of lactulose and mannitol postoral challenge. This allows calculation of a lactulose:mannitol ratio, which is a good marker of small intestinal permeability [45]. Intestinal permeability, measured by the lactulose:mannitol test, has been shown to be increased in autistic children compared to healthy controls [46–48]. Furthermore, co-localization of immunoglobulin G/complement C1q deposition on the basement membrane of epithelium of the GI tract is observed in ASD children, suggesting the presence of inflammatory processes and/or an autoimmune component that may affect the integrity of the mucosal barrier [41, 42]. It has been suggested that this increased permeability could lead to entering of byproducts of commensal or pathogenic bacteria as well as food-derived peptides into the blood. This entry of antigenic material may lead to immune responses that could affect neuronal signaling, or the material could directly interact with the peripheral nervous system [47, 48]. However, further testing is required [13], as the studies thus far have had methodological limitations including small subject populations. Properly powered prospective studies with appropriate controls are warranted to address the issues surrounding epithelial damage and permeability.

Probiotics are capable of stabilizing the mucosal barrier by increasing mucin expression, reducing bacterial overgrowth, stimulating mucosal immunity (secretory IgA), and synthesizing antioxidant substances [49]. Although most of these findings are based on cell culture systems and animal models, *in vivo* human work has also revealed enhanced expression of duodenal epithelial tight junction proteins in the context of short-term exposure to *L. plantarum* WCFS1 [50]. This supports the concept that probiotics can have a role in maintaining or improving gut barrier function in humans. In addition, specific probiotics have been proven to be successful in preventing the recurrence of inflammation in some situations and diseases such as IBD (for reviews see [51–53]). The controversies surrounding the extent and the nature of GI pathology in ASD exists, and studies that address the potential use of probiotics may well have to be confined to treatment of GI symptoms in ASD as opposed to clinically defined pathological criteria.

## 5. Immune Dysfunction

A possible role for immune dysfunction in ASD has been described for many years, and numerous immune abnormalities have been reported in ASD. However, results are rather variable and sometimes conflicting, due to the small number of study subjects, heterogeneous patient populations, and lack of proper controls [54]. Nevertheless, many findings point to aberrant immune activation, likely in a subset of individuals with ASD. These findings include an abnormal ratio of CD4^+^ to CD8^+^ T-cells, abnormal or skewed T-helper-cell (T_H_1/T_H_2/T_H_17) cytokine profiles, elevated blood monocyte counts, decreased lymphocyte numbers, self-reactive antibodies to brain and CNS proteins, neuroinflammation, imbalance of serum and mucosal immunoglobulin levels and increased nitric oxide mechanism (for reviews, see [39, 55, 56]). Immuno based therapies have largely been ignored in autism, but it has been shown that treatment with anti-inflammatory drugs induce clinical improvement in ASD [57].

A large part of the immune system (approximately 80%) is concentrated in and around the intestinal mucosa. The intestinal microbiota is involved in maturation of the immune system as demonstrated in studies in germ-free mice [58]. In turn, the microbiota in the intestines plays an important role in the regulation of functions in the immune system [59]. The immune system can be modulated by probiotic bacteria, and these effects are highly species and strain specific [11, 59, 60] with both *in vitro *cytokine responses and *in vivo* systemic effects altered by probiotics [61–67]. The accumulation of evidence suggests that probiotics play a role in providing tolerogenic signals. For example, a role for gut microbiota in autoimmune diseases has been suggested [68] and is supported by animal models demonstrating positive effects of probiotics on autoimmune diseases [66, 69, 70]. Although not much evidence has yet been reported on the effect of probiotics in autoimmunity in humans, a pilot study indicated positive effects in rheumatoid arthritis [71]. Probiotic bacteria themselves as well as metabolites produced by a probiotic bacterium have shown to suppress the activation of MCP-1, a chemokine shown to be dysregulated in ASD [72–75]. However, it must be noted that the effects of probiotics on cytokine and chemokine responses are highly strain specific, and probiotic bacteria differ in their ability to stimulate anti-inflammatory IL-10 secretion by peripheral blood mononuclear cells (PBMC) *in vitro *[76–78]. Bacterial strains with a good *in vitro *ratio of anti-inflammatory cytokines versus proinflammatory cytokines have been shown to have anti-inflammatory properties *in vivo *[76].* In vivo *administration of *Lactobacillus rhamnosus* GG induced higher IL-10 serum levels in allergic children [79], but this finding was not confirmed in other studies [64, 80]. Thus, while probiotics have good *in vitro* capacities to induce anti-inflammatory cytokines, their effect on systemic cytokine profiles remains to be proven and may be dependent on the microbiota mix *in situ*.

It is well established that there is bidirectional communication between the gut, the immune system, and the brain. For example, psychological stress can induce changes of the gastrointestinal microbes. On the other hand, intestinal bacteria can directly communicate with the central nervous system by the way of the vagal sensory nerve fibers and the peripheral immune system [81]. Intraventricular administration of propionic acid, a metabolite produced by gut bacteria, has been shown in a rat model to change both brain and behavior in a manner that is consistent with symptoms associated with ASD; these are not “autistic features” but may have some validity with ASD [82, 83]. It is possible that gut-brain interactions may contribute to abnormal neural development and the subsequent expression of aberrant behavior. Increased gut permeability may play an important role in the gut-brain relationship, as partially digested food and bacterial components can pass into the blood stream and may interfere directly and indirectly with the central nervous system. One of these immunoreactive compounds could be dietary-derived opioid peptides, derived from gluten and casein. Increased intestinal permeability can also permit entry of lipopolysaccharides (LPS), a potent proinflammatory compound of the cell walls of gram-negative bacteria. Leakage of lipopolysaccharides from the intestine might be the trigger for peripheral inflammatory responses that lead to *de novo* production of cytokines in the brain. By improving the epithelial barrier, this may reduce traffic of bacteria and their byproducts and might be a way to stop the inflammatory response. One study by Emmanuelle and colleagues showed increased LPS in the blood of individuals with ASD, a finding that corresponded to increased circulating IL-6 levels in the periphery and may reflect both an increased intestinal permeability and activation of immune responses that result in the production of this proficient neuromodulating cytokine [84]. 

Administration of probiotics can have influences on neuronal function, as shown by different studies. For example, administration of *Bifidobacterium infantis* in a rat model of depression showed effects on immune, neuroendocrine, and central monoaminergic activity [85]. The proinflammatory immune response was attenuated, and tryptophan was elevated by the bifidobacteria treatment. A probiotic drink containing *Lactobacillus casei *has also revealed positive effects on mood and cognition in volunteers [86]. Moreover, a pilot study in chronic fatigue patients has been performed with the same probiotic product [87]. Interestingly, almost all chronic fatigue syndrome patients report neuropsychological disturbances. The study showed a significant decrease in anxiety symptoms and suggested follow-up studies to examine anxiety and depression, including inflammatory cytokines and other immune mediators, blood tryptophan levels, and urinary metabolites of neurotransmitters [87]. Mechanisms of action by which probiotics can influence brain processes are not yet fully elucidated, but likely involve multiple pathways of the interplay between brain, gut, and immune system. Maintaining a balance between host defense and uncontrolled or unresolving inflammation relies on a mutualistic crosstalk between the immune system and the microbiome, especially in the maintenance of intestinal homeostasis, an area of relevance for a subset of ASDs [88]. Probiotics will become increasingly defined by strain-specific outcomes as this evolving area of cross-communication between the respective genes of the host and the microbiota becomes more clearly defined. Probiotics may offer a potential therapeutic that could beneficially alter the gut-brain axis and modify aberrant behaviors related to altered immune inflammatory outputs. 

## 6. Conclusions and Further Perspectives

Autism spectrum disorders are a diverse group of disorders caused by a complex interplay between genetic and environmental components. There is a range of indications that alterations in the intestinal microbiota in the gut might contribute to the disorder in a substantial number of individuals. Probiotics can be useful to restore the microbial balance in the intestine, to relieve gastrointestinal problems and to attenuate immunological abnormalities. Whether the use of probiotics by children with autism can lead to improvements in behaviors needs to be established in well-controlled trials with sufficient group sizes. Important for these trials is the choice of the bacterial strains, as effects of probiotic bacteria can be highly strain specific.
